# What rural residents want from blood donation services: a discrete choice experiment

**DOI:** 10.3389/fpubh.2026.1870500

**Published:** 2026-10-07

**Authors:** Latifat A. Bello, Oluwaseun A. Otekunrin, Olusegun S. Ewemooje, Kazeem A. Osuolale, Priscilla N. A. Ezemelue

**Affiliations:** 1 Department of Statistics, University of Ibadan, Ibadan, Nigeria; 2 Department of Statistics, University of Botswana, Gaborone, Botswana; 3 Grants Unit (Biostatistics), Nigerian Institute of Medical Research, Yaba, Lagos, Nigeria; 4 Nigerian Institute of Medical Research, Yaba, Lagos, Nigeria

**Keywords:** blood donation services, discrete choice experiments, health-related quality of life, Nigeria, rural residents’ preferences

## Abstract

**Background:**

Blood donation rates are low in Nigeria, and not much is known about rural residents’ preferences for blood services. Understanding what these residents want could lead to improved blood donation rates. This study aimed to determine rural residents’ preferences for attributes of blood donation services in Igbo-Ora, Nigeria.

**Methods:**

A thorough literature search and a focus group discussion with selected medical laboratory scientists were used to identify the attributes and their levels. A multi-stage sampling method was employed in selecting 150 respondents for the study. Nine choice sets were constructed using an efficient D-Optimal design, while a Discrete Choice Experiment was conducted using the nine choice sets with three hypothetical blood services in each set. Consistency of the respondents was measured by including the tenth choice set, which was a repetition of the first set. Parameter and willingness- to-pay estimates were obtained using mixed logit modelling.

**Results:**

The respondents’ mean age was 36.1 years, while 79.2% responded consistently to all choice set questions. Very low transportation costs and waiting time of within an hour for the blood donation process were preferred by the respondents. Free medical check-up was the most preferred attribute level, while the respondents showed a strong aversion to the meal voucher. Furthermore, attribute levels “donor only” and “anybody in need” were strongly disapproved. The respondents preferred their immediate family members to be recipients of blood donated by them.

**Conclusion:**

Rural residents preferred blood services with very low transportation costs, free medical check-ups as an incentive, and waiting time of within 1 h. These attribute levels deserve the attention of relevant policymakers in blood donation services.

## 1 Introduction

Blood is an important body fluid useful in transporting oxygen and nutrients to body cells and removing their metabolic wastes. It is made up of red blood cells, white blood cells, plasma, and platelets (1). Presently, there is no substitute for blood, making blood donation an important aspect of public health (2). Blood should be safe, in sufficient supply, and timely. These qualities are essential for saving lives and enhancing quality healthcare, especially in the African region with high rates of road traffic accidents and bleeding related to pregnancy and childbirth. Blood transfusions are also required for many other medical procedures, including surgeries, severe anemia, sickle cell disease, among others (3–5). Furthermore, blood has a very short lifespan. Thus, regular blood donation through volunteers is very important.

To be a blood donor, there are guidelines that must be strictly adhered to. According to the World Health Organization (WHO) standards, the usual age bracket for donating blood is between 18 and 65 years. First-time donors aged above 60 years should donate at a site where a medical doctor is available. An individual should weigh at least 45 kg to donate 350 mL ± 10% or 50 kg for 450 mL ± 10%. There are no upper weight limits, but gross obesity might lead to deferral due to other reasons, including inaccessible veins (6). In Nigeria, men and women are encouraged to donate blood once every three and 4 months, respectively, while donors should weigh at least 50 kg (7).

However, in many low- and middle-income countries, particularly in Nigeria, blood donation rates remain well below recommended thresholds, contributing to persistent shortages (8). The persistent low blood donation rates emphasize the need to understand the motivating factors and preferences of prospective blood donors in both rural and urban areas of Nigeria. Previous studies have focused mostly on knowledge, attitudes, and perceptions about voluntary blood donation in Nigeria. Misconceptions, cultural beliefs, limited access to information, and low exposure to health education have been identified as major contributing factors (9–11). However, studies on preferences for blood donation services are limited in Nigeria. Understanding what prospective blood donors want is critical for motivating, recruiting, and retaining more voluntary blood donors, especially in rural areas where access to blood donation services is limited. Therefore, this study aimed to identify attributes rural residents consider before participating in a blood donation exercise.

## 2 Methods

### 2.1 Discrete choice experiment

A DCE is a quantitative research technique widely used in measuring human preferences. It is usually employed to estimate the relative importance of the characteristics of a product or service, in conjunction with their trade-offs (12, 13). The DCE is rooted in consumer, random utility, and experimental design theories, and consists of sets of hypothetical choice tasks (scenarios) with options. Respondents are expected to rationally choose between two or more options in each choice task (12–14). In this study, three hypothetical blood services are presented in each choice set, and each respondent is expected to rationally choose an option from each set.

### 2.2 Attributes and levels

The authors conducted a thorough search of blood donation literature and identified relevant themes. A Focus Group Discussion (FGD) was then conducted with eight medical laboratory scientists working in selected secondary and tertiary public health facilities in Ibadan, the administrative capital of Oyo State. These medical laboratory scientists were selected because of the experience they have garnered over time interacting with blood donors from both rural and urban areas. Their experience, especially with blood donors from the rural areas, enabled a thorough discussion of the general themes identified in the literature. The attributes that were finally selected for the study were ‘waiting time’, ‘incentives’, ‘transportation costs’, and ‘access to donated blood’. These are presented in Table 1.

**Table 1:** Attributes and levels.

Attributes	Levels	Description
Waiting time	0	At most 1 h
	1	At most 2 h
	2	More than 2 h
Incentives	0	Meal voucher
	1	T-Shirts or Badges
	2	Free medical check-up
Transportation cost (in Naira)	0	200
	1	500
	2	700
Access to donated blood	0	Donor only
	1	Donor and immediate family members
	2	Anybody in need

### 2.3 Experimental design of the DCE

From Table 1, 81 treatment combinations $\left(3^{4}\right)$ would be required for the experiment, which was obviously not feasible. An efficient D-optimal design developed by Bush (15), with a smaller number of choice sets (nine sets), was adopted in this study. Each participant is expected to choose an option rationally from each of the choice sets. One of the choice sets is presented in Table 2.

**Table 2:** Example of a choice set.

Attributes	Service A	Service B	Service C
Waiting time	More than 2 h	At most 1 h	At most 2 h
Incentives	T-Shirts or Badges	Meal voucher	Free medical check-up
Transportation cost (in Naira)	₦200	₦500	₦700
Access to donated blood	Anybody in need	Donor or immediate family members	Donor only
Pick your choice here (please tick ONE box only)	▭	▭	▭

The D-optimal choice design estimates main effects by utilizing the multinomial logit (MNL) model. The mixed logit model was, however, used in this study because the effectiveness of the D-optimal choice design has been established for the multinomial logit and other models (16). Furthermore, the mixed logit model has the ability to overcome the limitations of the standard multinomial model and can also handle issues of several non-independent choices by the same respondent in DCEs (17, 18). Detailed information on the mixed logit model and the computation of the willingness-to-pay (WTP) estimates can be found elsewhere (17, 19–21).

### 2.4 Face validity and reliability

Face validity was determined by setting hypotheses about the levels of the attributes before the start of the experiment and confirming them through the signs of their estimates. The hypotheses were:

- Blood donors prefer a service where the waiting time is within 1 h.
- Blood donors prefer free medical check-up to any other type of incentives.
- Blood donors prefer very low transportation costs.

The first choice set was repeated and included as the tenth choice set to check respondents’ choice consistency. The percentage of participants with consistent responses was calculated.

### 2.5 Study area

The study was purposively conducted in Igbo-Ora, a town situated 80 km north of Lagos, southwest Nigeria. It is the headquarters of Ibarapa Central Local Government of Oyo State. Historically, the community has seven quarters, namely Iberekodo, Igbole, Pako, Sagan-un, Lagbaye, Idofin and Igbo-Ora itself (22). The community, with an estimated population of about 92,719 people, is mostly rural, predominantly Yoruba, with the majority of the inhabitants being peasant farmers and artisans (23, 24).

### 2.6 Sample size determination and sampling procedure

Based on the rule of thumb provided by Johnson and Orme (25), the sample size was estimated using $\frac{\mathrm{nta}}{c}\geq 500$ where n= number of participants, t=9 is the number of choice sets, a=3 is the number of alternatives, and c=3 is the largest number of levels for any of the attributes. This gives n=56 but was increased to 150 to ensure compliance with model estimation rules (12, 18, 26).

A multi-stage sampling technique was used. Firstly, 15 clusters were identified in the community using identifiable landmarks. Secondly, from each cluster, 10 households were randomly selected, and lastly, one individual was selected from eligible adults in each household, giving a total of 150 respondents.

### 2.7 Inclusion and exclusion criteria

Inclusion: Healthy adults who were at least 18 years old.

Exclusion: Non-Yoruba-speaking adults, pregnant women, those who were ill, or those with cognitive disability who were unable to answer questions reliably and consistently.

### 2.8 Ethical approval and informed consent

Ethical approval for the study was granted by the Oyo State Ministry of Health Research Ethics Committee with reference number AD 13/479/712^A^.

A written informed consent form was filled and signed by consenting participants before the administration of the questionnaire.

### 2.9 Measurement instruments

Relevant socio-demographic, knowledge, willingness to donate blood, and the practice of blood donation data were collected using a structured interviewer-administered questionnaire. Assessment of respondents’ physical and mental health status was also conducted using the validated Yoruba version of the Short Form-12 Health Survey (Y-SF-12). Furthermore, a DCE was conducted to identify preferred attributes and levels of hypothetical blood donation services.

### 2.10 The Yoruba short form-12 health survey

The Y-SF-12 is a validated Yoruba version of the SF-12 Health Survey developed by Mbada et al. (27). It is used to measure overall physical and mental health outcomes in general Yoruba populations. The SF-12 Health Survey is a comprehensive, self-reported, and widely used Health-Related Quality of Life (HRQoL) questionnaire derived from the 36-Item Short Form Survey. It was developed especially for use in large surveys to overcome challenges of respondent burden and data collection management associated with the lengthy 36-Item Short Form Survey (28, 29). The SF-12 has two summary scores: the Physical Component Summary (PCS) and the Mental Component Summary (MCS). It has been successfully utilized in hospitals and public health research settings (27–30). A freely available web-based scoring tool^1^ for the non-proprietary SF-12 (version 1.0) was used to compute the PCS-12 and MCS-12 scores for the Y-SF-12 in this study.

### 2.11 Questionnaire administration and data analysis

The questionnaire was divided into four sections, namely:

- socio-demographic,
- knowledge, willingness to donate blood, and the practice of blood donation
- Y-SF-12, and
- choice sets

The socio-demographic, knowledge, willingness to donate blood and the practice of blood donation, and the choice sets were also translated into the Yoruba language by the authors to facilitate easy comprehension by the participants. A formal back-translation protocol was followed to ensure the technical terms of the DCE remained consistent across both English and Yoruba languages. The pilot testing of the instruments was conducted using 10 respondents who were not part of the sampled population. A comprehensive review of the questionnaire was conducted to remove all forms of ambiguity based on reports received from the pilot study before final questionnaire administration. The questionnaires were administered using a face-to-face interview method to ensure that every section was completely filled. Statistical analysis was carried out using Stata (Release 14.2, StataCorp LLC, College Station, TX).

## 3 Results

### 3.1 Socio-demographic profile

The response rate was 100%. The *a priori* hypotheses set had expected signs confirming the face validity of the study, while 79.2% made their choices in a consistent manner.

The mean age of the respondents is 36.1 years, while the mean household size is 6.5 with minimum and maximum household sizes being 1 and 20, respectively. Most of the respondents are females (56%), with the majority of these females (67.9%) in the “18 - 39” years category. More than half of the respondents (56.7%) have no formal education. The mean monthly income of the respondents is ₦61,900, while the minimum and maximum values are ₦7,000 and ₦350,000, respectively. More females (69.4%) earn below ₦40,000 compared to males, while more males (50.7%) earn between ₦40,000 and ₦80,000 compared to females. Furthermore, 63.3% of the respondents are married or cohabiting, while 62.1% of them are from monogamous families (Table 3).

**Table 3:** Respondents’ Socio-demographic Information.

Variable	Frequency	Percent (%)
Gender		
Female	84	56
Male	66	44
Age		
18–39	90	60
40–61	51	34
62 and above	9	6
Level of education		
None	85	56.7
Secondary education or less	50	33.3
Tertiary	15	10.0
Religion		
Christianity	73	48.7
Islam	70	46.7
Others	7	4.7
Marital status		
Single/separated	51	34.0
Married/cohabiting	95	63.3
Widowed	4	2.7
Family type		
Monogamous	90	60
Polygamous	60	40
Occupation		
Farming	94	62.7
Trading	39	26.0
Others	17	11.3
Household size		
1–4	53	35.3
5–8	49	32.7
9 and above	48	32.0
Monthly income (in Naira)		
Less than 40,000	49	32.7
40,000–80,999	73	48.7
81,000 and above	28	18.7

### 3.2 Respondents’ self-perceived health status

The mean PCS and MCS scores were 47.25 ± 8.66 and 45.93 ± 7.07, respectively. Half (50%) of the female respondents had above 50 PCS score, indicating a physical health status that is above average. However, 51.5% of the males had PCS scores below 50, indicating a poorer physical health status. For the MCS scores, 76.2 and 69.7% of the females and males, respectively, had scores below 50 suggesting poorer mental health status among the respondents. The self-perceived health status of the respondents is presented in Table 4.

**Table 4:** Self-perceived health status of the respondents.

Score	Mean (SD)	Respondents with scores below the mean [number (%)]	Respondents with scores above the mean [number (%)]
PCS	47.25 (±8.66)	76 (50.7)	74 (49.3)
MCS	45.93 (±7.07)	110 (73.3)	40 (26.7)

### 3.3 Blood donation knowledge, willingness to donate blood and practice of donating blood

About 66.7% have heard about blood donation, while above 70% do not know the required age, minimum weight, standard amount, and frequency of blood donation in Nigeria. Furthermore, 72.7% of the respondents expressed their willingness to donate blood, while 18% have donated blood before. There is a significant association between the level of education of the respondents and previous blood donation (Tables 5, 6).

**Table 5:** Blood donation knowledge, willingness and practice.

Knowledge variables	Frequency (%)
Have you heard about blood donation?	
Yes	100 (66.7)
No	50 (33.3)
Minimum age required for blood donation	
<18 years	14 (9.3)
>18 years	14 (9.3)
18 years	14 (9.3)
I do not know	108 (72.0)
Minimum weight required for blood donation	
<50 kg	10 (6.7)
≥50 kg	4 (2.7)
≥45 kg	6 (4.0)
I do not know	130 (86.7)
Frequency of blood donation	
Once in 3 months	8 (5.3)
Once in 6 months	12 (8.0)
Once in 1 year	17 (11.3)
I do not know	113 (75.3)
Minimum blood quantity that can be donated in Nigeria	
450 ml	25 (16.7)
500–1,000 mL	6 (4.0)
I do not know	119 (79.3)
Willingness to donate blood	
Yes	109 (72.7)
No	41 (27.3)
Previous Blood Donation	
Yes	27 (18.0)
No	123 (82)

**Table 6:** Socio-demographic variables according to previous blood donation.

Variable	Previous blood donation (%)		$\chi^{2}$	*p*-value
	Yes	No		
Gender				
Female	11 (13.1)	73 (86.9)	3.0923	0.079
Male	16 (24.2)	50 (75.8)		
Age				
18–39	18 (20.0)	72 (80.0)		0.635
40–61	7 (13.7)	44 (86.3)		
62 and above	2 (22.2)	7 (77.8)		
Income (in Naira)				
Less than 40,000	9 (18.4)	40 (81.6)	0.3409	0.843
40,000–80,999	12 (16.4)	61 (83.6)		
81,000 and above	6 (21.4)	22 (78.6)		
Occupation				
Farming	14 (14.9)	80 (85.1)	3.5141	0.173
Trading	7 (18.0)	32 (82.1)		
Others	6 (35.3)	11 (64.7)		
Family type				
Monogamous	19 (21.1)	71 (78.9)	1.5206	0.218
Polygamous	8 (13.3)	52 (86.7)		
Level of education				
No education	11 (12.9)	74 (87.1)	8.0556	0.018*
Secondary education or less	9 (18.0)	41 (82.0)		
Tertiary	7 (46.7)	8 (53.3)		
Marital status				
Single/separated	8 (15.7)	43 (84.3)		0.707
Married/cohabiting	18 (19.0)	77 (81.1)		
Widowed	1 (25.0)	3 (75.0)		

*significant at 5%.

### 3.4 Respondents’ preferences for attributes of Mobile blood donation services

The coefficient of “transport cost” is negative and significant, indicating that respondents strongly disapprove of high transportation costs. The coefficient of “at most 1 hour” is positive and significant. This implies that, on average, respondents preferred waiting at most 1 h compared to waiting for more than 2 h. However, the coefficient of “at most 2 hours” is positive but not significant. The “meal vouchers” coefficient is negative and significant, indicating that respondents strongly dislike collecting meal vouchers as an incentive before blood donation. The coefficient of “free medical check-up” had the strongest influence on the probability of selecting a blood donation service ( β=1.0166) and it is the most preferred of all the attribute levels. Furthermore, the coefficients of the levels “donor only” and “anybody in need” are negative and significant, indicating that the respondents preferred blood donation services that provide future access to blood for the donor and immediate family members. Of all the attribute levels, “meal voucher” is the least preferred (β=−0.4710) (Table 7).

**Table 7:** Respondents’ preferences for attributes of blood donation services.

Variable	Coeff.	Std. Err.	P>∣Z∣
Transport cost	−0.0008	0.0003	0.003
SD	0.0022	0.0003	0.000
At most 1 h	0.3699	0.1165	0.001
SD	0.8424	0.1268	0.000
At most 2 h	0.1230	0.0933	0.187
SD	−0.0118	0.1779	0.947
Meal voucher	−0.4710	0.1816	0.009
SD	1.6258	0.1756	0.000
Free medical check-up	1.0166	0.1980	0.000
SD	2.3109	0.2247	0.000
Donor only	−0.4467	0.0931	0.000
SD	−0.0946	0.2630	0.719
Anybody in need	−0.2590	0.1011	0.010
SD	0.6268	0.1329	0.000

Number of respondents = 150; Coeff.: coefficient; Std. Err.: standard error; C. I.: confidence interval; log likelihood = −1140.472; likelihood ratio $\chi^{2}(7)=533.46$ ; probability $>\chi^{2}$ = 0.000.

### 3.5 Respondents’ WTP for attributes of a blood donation service

The results are presented in Table 8. The mean WTP for ‘meal voucher’, ‘donor only’ and ‘anybody in need’ are negative and significant, indicating respondents’ strong aversion to these levels. Furthermore, the attribute level “free medical check-up” increases WTP by ₦1312.625.

**Table 8:** Respondents’ WTP for attributes of a blood donation service.

Variables	Mean WTP estimates (N)	95% C. I.
Waiting time		
At most 1 h	477.645	(59.732, 895.558)*
At most 2 h	158.809	(−100.383, 418.001)
Incentives		
Meal voucher	−608.186	(−1,210.598, −5.774)*
Free medical check-up	1312.625	(349.178, 2,276.072)*
Access to donated blood		
Donor only	−576.769	(−1,016.240, −137.299)*
Anybody in need	−334.378	(−665.232, −3.523)*

C.I.: confidence interval; *Confidence interval does not include zero.

## 4 Discussion

More than half of the respondents (56.7%) had no formal education. This contradicts Verma et al. (31), who reported a lower percentage (23.8%) among Indian rural adults aged 18–59 years in their study. This could be attributed to the different literacy levels in both countries. The literacy level (percent of people aged 15 and above), as of 2024, for Nigeria and India is 70 and 78%, respectively (32).

The self-perceived health status of the respondents revealed that more than half (PCS: 50.7%; MCS: 73.3%) of the respondents had a poorer-than-average HRQoL. Poorer-than-average HRQoL was also reported for both rural and urban populations in an SF-12 Health Survey study conducted by Ibrahim et al. (29) in Nigeria.

More than half (66.7%) have heard about blood donation. Despite the high level of awareness, few of the respondents (18%) have previously donated blood, implying that awareness does not necessarily translate to blood donation. In this study, more than 70% of the respondents had poor knowledge of blood donation practices, and this could be one of the factors that contributed to the low blood donation rates. Salaudeen et al. (33) also reported poor knowledge about blood donation practices and suggested the dissemination of information about blood donation practices during awareness campaigns. Gbadamosi et al. (34) emphasized the need for improved knowledge about blood donation among rural dwellers. A significant association between the level of education of the respondents and previous blood donation further corroborated this assertion and agreed with a previous study conducted by Ogundeji et al. (35).

Waiting time is one of the attributes considered in this study. The respondents preferred “at most 1 hour” compared to the “more than 2 hours” attribute level. They were willing to pay an extra ₦444.65 to get this attribute level in their preferred blood service. The American National Red Cross (36) reported that it takes up to an hour for the entire process to be completed from the donor’s time of arrival. The WHO and International Federation of Red Cross and Red Crescent Societies (IFRC) (37) have also identified long waiting times and a lengthy donation process as one of the factors hindering blood donation.

The respondents were averse to the offer of meal vouchers as incentives for blood donation. This is similar to results obtained by Mohammed et al. (38) and Finck et al. (39). However, free medical check-up was the most sought-after attribute level by the respondents. This is consistent with the result obtained by Leipnitz et al. (40), which showed that a thorough blood health check incentive had a positive influence on blood donation behaviour.

The desire to help immediate family members was the main motive behind blood donations by the respondents. They preferred to donate blood with the condition that the donor and immediate family members would have access to the donated blood. This is consistent with a study conducted by Mohammed and Essel (38), which reported that donors were not interested in helping anybody in need, but rather their immediate family members and friends.

### 4.1 Study limitations

This study has two major limitations. Firstly, only Igbo-Ora, Oyo State, Nigeria was used as the study site. Thus, extra caution should be taken when generalizing results obtained to all rural residents in Nigeria. This is because the study did not capture the distinct characteristics of all rural residents in every region of Nigeria. Secondly, real choices of the participants were not determined due to limitations associated with conducting real experiments. This limitation can be overcome by including qualitative studies in similar experiments in the future.

## 5 Conclusion

In this paper, rural residents’ preferences for attributes of blood services were determined. These residents preferred very low transportation costs to blood donation centres. They also preferred waiting times for the blood donation process that do not exceed 1 h. Furthermore, they preferred free medical check-ups to meal vouchers, gifts such as T-shirts, badges, and so on. Lastly, they preferred their immediate family members to be recipients of blood donated by them. These attribute levels deserve the attention of relevant policymakers in blood donation services so that blood donation rates can improve in Nigerian rural areas.

## Data Availability

The original contributions presented in the study are included in the article/supplementary material, further inquiries can be directed to the corresponding author.

## Footnotes

1
www.orthotoolkit.com/sf-12/
